# Heritage Community Resilience: towards new approaches for urban resilience and sustainability

**DOI:** 10.1186/s40410-020-00126-7

**Published:** 2020-11-11

**Authors:** Katia Fabbricatti, Lucie Boissenin, Michele Citoni

**Affiliations:** 1 Department of Architecture DiARC, University of Naples “Federico II”, Via Tarsia 31, 80135 Naples, Italy; 2 LabEx Architecture, Environment and Building Cultures, Grenoble School of Architecture, Univ. Grenoble Alpes, 60 avenue de Constantine, 38036 Grenoble, France; 3 ACT Antropologia Cultura Territorio, Via Festo Avieno 230, 00136 Rome, Italy

**Keywords:** Cultural heritage, Heritage community, Community resilience, Inner peripheries, Cultural and creative practices, Urban resilience

## Abstract

The value of cultural heritage and its transmission for “making cities and human settlements inclusive, safe, resilient and sustainable” is an integral part of the UN Agenda 2030 and the new international policy for Disaster Risk Reduction 2015–2030. Nonetheless, the role of culture for these important challenges is an issue that current scientific literature on resilience has not yet sufficiently investigated. Starting from the concept of Heritage Community, elaborated in the Council of Europe's Framework Convention on the Value of Cultural Heritage for Society (Framework convention on the value of cultural heritage for society, 2005), along with the hypothesis of its role for Community Resilience, this study elaborates a conceptual framework in which “Heritage Community Resilience” is defined. It is both a target and a process in which cultural heritage supports the building of a community able to prevent, cope with and recover from disturbances and/ or disasters. Through a survey of several case studies on heritage-driven practices in Italian inner peripheral areas, the research aims to define the specific characteristics of Heritage Community Resilience as well as identify any critical actors and variables, strategies and governance mechanisms, which influence both Heritage Community and Community Resilience. It predicts the challenges and highlights the potential that culture and heritage can develop for Community Resilience, towards further perspectives of resilient circular city.

## Introduction

Cities now have to face various types of risks, including frequent or infrequent events, with either sudden or slow-onset natural or man-made hazards, that can occur both globally (climate crisis, scarcity of resources, migrations, etc.) and locally (earthquakes, depopulation, erosion of cultural capital, etc.). Urban resilience is an international topic of discussion (Pu and Qiu 2016) in global policy frameworks (UNESCO 2013, 2015; UNDRR 2015a; UNDRR 2015b). Nonetheless, urban resilience still has a low profile in some policy arenas (Chmutina et al. 2016), with cities being far from reaching the goals set by international programmes or standards, as the current COVID-19 pandemic seems to confirm at a global scale, even though the available data are still partial and contradictory.

In the governance debate, Community Resilience is developing into an autonomous theme (Coaffee 2013; Mulligan et al. 2016; Chelleri et al. 2016). It is becoming a target for society development for both scientific literature (Berkes and Ross 2013; Chaskin 2008; Cutter et al. 2008; Maguire and Cartwright 2008; etc.) and National Security Policies (UK Community Resilience Programme, US Community Resilience Taskforce, Australian National Strategy for Disaster Resilience, etc.). In recent years, communities are demanding and gaining autonomy to address deficiencies in development policies. The community is an integral part of social-urban and socio-ecological systems, with it represents the most dynamic part of them. Therefore, resilience must be pursued on the community scale (Longstaff et al. 2010).

Recent global policy documents (UNESCO 2013, 2015; UNDRR 2015a; UNDRR 2015b) link Community Resilience to the strengthening of culture and cultural heritage. For the first time in 2013, cultural heritage was recognised as an important “actor” in addressing global risks, especially for its role in strengthening CR (UNESCO 2013, action 6). The session “Resilient Cultural Heritage” of the Third United Nations World Conference on Disaster Risk Reduction (WCDRR) (UNDRR 2015b) stressed how any Disaster Risk Reduction policies and programmes should consider the cultural context, including cultural heritage as its most symbolic manifestation, if it is to be effective and sustainable. In resilience debates, however, this is an issue that current literature has largely failed to contemplate (Beel et al. 2017).

The Council of Europe's Framework Convention on the Value of Cultural Heritage for Society (Council of Europe 2005, art. 2b) shifts attention from the cultural heritage in itself towards people and their active participation in the process of recognizing the values held in it and their transmission to future generations (Council of Europe 2018). It defines “Heritage Community” as a community that “values specific aspects of cultural heritage which it wishes, within the framework of public action, to sustain and transmit to future generations” (Council of Europe 2005, art. 2b). A Heritage Community is characterized by awareness of the resource value of its cultural heritage, a sense of belonging, inclusiveness, collaboration at all levels, a common interest in heritage-led actions, shared civic responsibility towards cultural heritage. Based on the role of culture and cultural heritage in relation to Community Resilience, this study elaborates a conceptual framework in which “Heritage Community Resilience” is considered as being both a target and a process in which cultural heritage supports the building of a community able to prevent, cope with and recover from disturbances. In an evolutionary vision of resilience, in which communities do not return to an initial state but evolve (Davoudi et al. 2012; Pink and Lewis 2014; Hillier 2015; etc.), culture and cultural heritage can be the key to citizen engagement (idea of common good), as well as to social, environmental, economic and governance innovation.

Through a survey of several case studies on heritage-driven practices, the research aims to: define the specific characteristics of Heritage Community Resilience; identify the effects of the practices for building Heritage Community and Community Resilience processes; define critical actors and variables, strategies and governance mechanisms, which influence both HC and CR.

European peripheral areas, with particular attention to Alta Irpinia in southern Italy, represent the research context. Here, recognition and promotion of culture and creativity are particularly critical challenges. In these contexts, culture and creativity can help maximise economic returns from the production of place-specific and high-value-added products, favouring citizen engagement and community building (European Commission 2017).

From the lessons learned in the case studies in Alta Irpinia, the research predicts the challenges and highlights the potential that culture and heritage can develop for Community Resilience.

The paper is articulated as follows: first, the concept of CR is discussed within the international debate on resilience, with a focus on culture and cultural heritage; then the conceptual framework of the research is presented; in the second section, the survey method of the case studies is defined and then the research context is presented; finally, the cases are described and discussed.

## Literature review

Community Resilience is an increasing target for social and urban development, as scientific literature (Berkes and Ross 2013; Chaskin 2008; Cutter et al. 2008; Maguire and Cartwright 2008; Sharma and Verma 2019; etc.), international documents (UNESCO 2013, 2015; UNDRR 2015a; UNDRR 2015b) and some agency and state strategies declare (Bach 2015; Mulligan et al. 2016).

The assumption of a social component, within the original ecological resilience approach, started in the late 1990s, with a progressive enrichment of both social and ecological domains. “The social-ecological understanding of resilience emerged with the assumption that social and ecological systems were inextricably interconnected and that local communities could be made more resilient to unexpected shocks if efforts were made to increase their adaptive capacity” (Mulligan et al. 2016, p. 3). The latter is a property of the social–ecological system, with it distinguishing humans from animals and plants; the former can anticipate change and together with social, political and cultural experiences influence resilience (Folke et al. 2010). Communities do not control all of the conditions that affect them, but they can change some of the conditions that can increase their resilience (Berkes and Ross 2013).

As some authors have pointed out (Mulligan et al. 2016; Berkes and Ross 2013; etc.), although the approach to resilience significantly benefited from social-ecological cross-fertilization, the word “community” continued to be used rather uncritically. The causes highlighted by the authors are related to the socio-ecological approach, developed in fields such as biophysical or environmental sciences and resource economics rather than in sociological or cultural studies.

A significant contribution to the evolution of the concept of Community Resilience comes from the field of psychology. In Berkes and Ross’ research on the Mental Health and Developmental Psychology (Berkes and Ross 2013), resilience is defined as a process of dynamic personal development in the face of adversity and adaptation, rather than as a stable outcome that is achieved and then retained (Luthar and Cicchetti 2000; Almedom et al. 2007). The extension of this study to the community scale focuses on identifying the strengths of a community, and how they contribute, in a collective process, to facing challenges and developing resilience (Kulig et al. 2008; Berkes and Ross 2013; Norris et al. 2008; Buikstra et al. 2010; etc.). Several studies agree that the main strengths of a community are: individual psychological components—social networks, social inclusions, sense of belonging, leadership, outlook on life, learning (Norris et al. 2008; Kulig et al. 2010; Buikstra et al. 2010; etc.), the natural and built environment they are aware of, the lifestyles and livelihoods, where the role of infrastructure and support services are particularly important in disaster recovery (Kulig et al. 2008). In applied uses of psychological resilience thinking, it is important to highlight how human beings can train resilience through their responses to shocks and stresses, and actively develop resilience through capacity building and social learning (Goldstein 2009).

In the evolution of the issue of Community Resilience, scholars agree that some of the greatest progress has been made in urban planning, bringing together community and resilience in a meaningful way. In the urban context, as a complex adaptive social-ecological system, “a sophisticated understanding of both socio-ecological systems and the more cultural and political conceptions of the community” emerge (Mulligan et al. 2016, p. 5).

In the field of urban studies, the evolutionary perspective of resilience (Davoudi et al. 2012; Pink and Lewis 2014; Hillier 2015) is “understood not as a fixed asset, but as a continually changing process; not as a being but as a becoming” (Davoudi et al. 2012, p. 304). To the interpretation of resilience as “dynamic interaction of persistence, adaptability and transformability on multiple scales” introduced by Folke et al. (2010) a fourth component “preparation”, based on learning ability has been added, which reflects “the intentionality of human action and intervention” (Davoudi et al. 2012). This is typical of social systems, enhancing the key role of social capital and institutions in the building up of resilient cities (Galderisi 2018).

The ability of people to learn from experience, to increase their abilities to prepare, cope with and recover from disturbances is therefore the basis for Community Resilience. Bulley (2013) noted that communities need to be ‘produced’ before they can be mobilized. Rather than a static and vulnerable entity, communities are therefore as complex assemblages ‘making’ resilience at, across and between local and global scales (Pink and Lewis 2014). Community Resilience is therefore not a target but a dynamic process based on continuous learning (Cutter et al. 2008; Wilson 2014).

In recent years, strengthening Community Resilience has emerged as an essential element of National Security Policies to address climate change and risks (Bach 2015; Mulligan et al. 2016). In 2008, the UK Government launched in 2008 a CR Programme and published in 2011 the Strategic National Framework on CR, which “is intended to provide the national statement for how individual and community resilience can work”, and “should be relevant to all hazards and threats, and all communities” (UK Cabinet Office 2011).

In the USA, the Federal Emergency Management Agency considers Community Resilience as one of the “core capabilities” needed to achieve the “preparedness” for all types of disasters and emergencies (FEMA 2015). The entire strategy is based on a “whole community” approach (FEMA 2015), which aims to engage society at all levels: “The core value proposition of this whole community approach is that by strengthening the assets, capacities, relationships and institutions within a community before disasters strike the community will prepare more effectively, better withstand the initial impacts of an emergency, recover more quickly, and adapt to become better off than before the disaster hit” (Kaufman et al. 2015, p. 158). Some other examples are worth mentioning, like the Australian National Strategy for Disaster Reduction, 2011, that of New Zealand after Christchurch earthquake, and the Netherlands which experimented a “living with the water” approach that involved a public–private cooperation to face the growing climate threats (Goemans et al. 2015). As Mulligan noted, these national Community Resilience policies and programmes assume that more resilient local communities will make for a more resilient national community (Mulligan et al. 2016).

Some of the above mentioned strategies, such as the Australian and US, point to cultural heritage as one of the assets that people prepare to protect in a disaster resilient community. However, there is still little reference in these programs to the role of cultural heritage in building Community Resilience. Indeed, only recently, the debate on resilience has been enriched by the emerging issues related to culture and cultural heritage. In particular, the topic is addressed in some global policy documents (UNESCO 2013, 2015; UNDRR 2015a) and is being discussed in further detail in current scientific literature (Jigyasu 2013; Holtorf 2018).

In 2013, for the first time in international documents, cultural heritage was recognized as playing a role in addressing global risks, especially for its ability to strengthen Community Resilience (UNESCO 2013). In the Hangzhou Declaration, Placing Culture at the Heart of Sustainable Development Policies, “the appropriate conservation of the historic environment, including cultural landscapes, and the safeguarding of relevant traditional knowledge, values and practices, in synergy with other scientific knowledge, enhances the resilience of communities to disasters and climate change” (UNESCO 2013, action 6). Later, during the Third United Nations World Conference Disaster Risk Reduction (WCDRR), in which the Sendai Framework for DRR 2015–2030 was adopted, it was recognized that “cultural heritage provides important insights and opportunities for enhancing Disaster Risk Reduction, post-disaster rehabilitation and recovery, building back better and for stimulating local economic and social development» (UNDRR 2015a). The session on “Resilient Cultural Heritage” highlighted how any Disaster Risk Reduction policies and programmes should consider the cultural context, including cultural heritage as its most symbolic manifestation, to be effective and sustainable (UNDRR 2015b).

In the 2030 Agenda for Sustainable Development (UNESCO 2015) and the New Urban Agenda (UN 2016), culture emerges as a transversal driver, both as a knowledge capital and source of creativity and innovation, as well as a resource to face challenges and find appropriate solutions. “Culture is who we are, and what shapes our identity. Placing culture at the heart of development policies is the only way to ensure a human-centred, inclusive and equitable development” (Hosagrahar et al. 2016). In the Document of the UN Conference on Housing and Sustainable Urban Development Habitat III (UN 2016), it is stated that culture allows to revitalize urban areas, strengthens social participation (point 38) and contributes to developing vibrant, sustainable and inclusive urban economies (points 45 and 60).

In scientific debates, the question of the relationship between heritage and resilience is spreading and evolving, although the debate is still sectoral (Beel et al. 2017). In most cases, the issue is addressed starting from the need to safeguard cultural heritage, recognizing its important role for the well-being and quality of life of people (Azadeh et al. 2015; etc.). Some studies carried out as a result of the analysis of the processes that occurred before and after the disasters, have highlighted the contribution of the local material culture in prevention and recovery from risks. In the prevention phase, for example, the role of knowledge of traditional construction techniques or traditional prevention strategies resulting from subsequent trial and error in the management of known and expected risks is underlined (Jigyasu 2013; D’Amico and Currà 2014; Boccardi 2015; etc.).

A recent paper by Holtorf (2018), UNESCO Chair on Heritage Futures in Sweden, suggests an approach to Cultural Resilience (Crane 2010) in which cultural heritage promotes resilience “precisely through the way, often highly evident, in which it has been able to adapt and develop in the past” (Holtorf 2018, p. 647). In this article, the author suggests that cultural resilience, risk preparedness, post-disaster recovery and mutual understanding between people will be better enhanced by a greater capacity to accept loss and transformation. In the author's view, the visible changes in cultural heritage over time can inspire people to embrace uncertainty and absorb adversity in times of change, thus increasing their cultural resilience (Holtorf 2018).

The present research proposes new elements for the debate on the role of cultural heritage for resilience, and in particular for Community Resilience, with the concept of “Heritage Community Resilience”. The paper focuses on the capacity of cultural heritage to make and innovate communities (Council of Europe 2005), in a proactive process aimed at preventing, coping with and recovering from disturbances and/ or disasters.

## Conceptual framework

The Framework Convention on the Value of Cultural Heritage for Society (Council of Europe 2005) marks a revolution in the meaning of cultural heritage, shifting the attention from objects and places to people: “cultural heritage is a group of resources inherited from the past which people identify, independently of ownership, as a reflection and expression of their constantly evolving values, beliefs, knowledge and traditions. It includes all the aspects of the environment resulting from the interaction between people and places through time” (art. 2a).

This new way of looking at heritage lays the foundations for redesigning relations between all the involved stakeholders. It stresses the crucial role of inhabitants and, as suggested by the Convention, of a real “Heritage Community” (art. 2b). The Faro Convention approach empowers communities to take an operational role in decision-making towards direct democracy as well as contribute to the defining of policies and strategies regarding their local resources. Heritage Communities are defined as “people who value specific aspects of cultural heritage which they wish, within the framework of public action, to sustain and transmit to future generations” (art. 2b). In the aim of the Convention, Heritage Communities are self-organized and self-managed groups of individuals interested in a progressive social transformation of relations between peoples, places and histories. They have an inclusive approach based on a better definition of heritage.

The principles upon which the Convention is based are listed in the Action Plan:

- “Connection to a community and territory determines a sense of belonging;
- Social cohesion is founded on various levels of cooperation and commitment;
- Democracy is practised through the engagement of civil society in dialogue and action, through shared responsibilities based on capacities” (Council of Europe 2018, p. 6).

The Convention thus marks a definitive passage from the “right *of* cultural heritage” to the “right *to* cultural heritage”, progressing from a static idea of the “value in itself” of cultural heritage towards a proposition of “relational value”, which links in an interactive, dynamic and complex way “people and places through time” (art. 2b). Heritage Communities are the testimony and vehicle of local identity values to preserve and transmit to future generations. They are also “cultural” laboratories, drivers of inclusive actions, collaboration at all levels, of heritage-led actions, shared civic responsibility towards cultural heritage.

For the purposes of this research, the attributes of a HC can be summed up in: awareness of the value of its cultural heritage resources, sense of belonging, inclusiveness, collaboration at all levels, common interest in heritage-driven actions, shared civic responsibility towards cultural heritage (Council of Europe 2018).

The research hypothesises that these attributes can support Community Resilience at different stages of its life (Asprone and Manfredi 2015), and in particular in Disaster Risk Reduction. In this hypothesis, the research develops a conceptual framework in which it defines “Heritage Community Resilience”. It represents both an objective and a process in which the community builds, through cultural heritage, its capacity to anticipate and adapt to the challenges and stress factors encountered before, during and after a disaster and/or disruption.

In the prevention and protection phase, Heritage Community Resilience is characterized by strength in terms of identity and recognisability, ethics, knowledge; it acts in reducing vulnerabilities through community care and maintenance of cultural heritage; it is also characterized by creative and innovative strategies and policies for Disaster Risk Reduction. In the reaction and recovery/adaptation phases, HCR is characterized by a sense of belonging that is a powerful catalyst for the involvement of the local population; it allows for rapid recovery through income generated in the informal sector and in tourism activities, through creative and innovative cultural adaptation solutions.

This new vision of community entrusts a shared responsibility of all the actors towards heritage, implicitly imposing shared policies among institutions, sector experts, national authorities (D’Alessandro 2015). This is a fundamental principle of “cultural democracy” (Ibidem, p. 82), which transfers responsibility to the same subjects that determine the meaning and value of the patrimonial elements with which they identify.

Recent global policy frameworks underline that the assumption of cultural heritage in Disaster Risk Reduction requires enlargement and differentiation of the arena of actors, towards innovative partnerships between the heritage sector, on one hand, and the wide range of DRR stakeholders, including local governments, humanitarian organizations and the private sector (UNDRR 2015a). It is underlined also the role of educational and research institutions in supporting the various actors in the different phases (UNDRR 2015a, art. 36b).

On these bases, this paper aims to identify the critical actors and variables, strategies and governance mechanisms that influence Heritage Community Resilience, in a self-sustaining circuit in which Heritage Community care actions can reduce the vulnerability of cultural heritage and community, and at the same time increase its capacity to prevent, cope with and recover from disturbances and/or disasters.

## Materials and methods

The research method was a case study survey. The objectives of the survey were to elaborate in greater detail the characteristics of Heritage Community Resilience, to define the effects of the practices for building related Heritage Community and Community Resilience processes, identify the critical actors and variables, strategies and governance mechanisms influencing HCR. For these purposes, the research analysed heritage-driven as well as both bottom-up or mixed bottom-up and top-down practices.

The method was based on direct surveys, carried out through widely distributed questionnaires and interviews with selected stakeholders. These surveys were conducted through a previous definition of the Heritage Community Resilience indicators that guided the collection and interpretation of data.

**Fig. 1:**
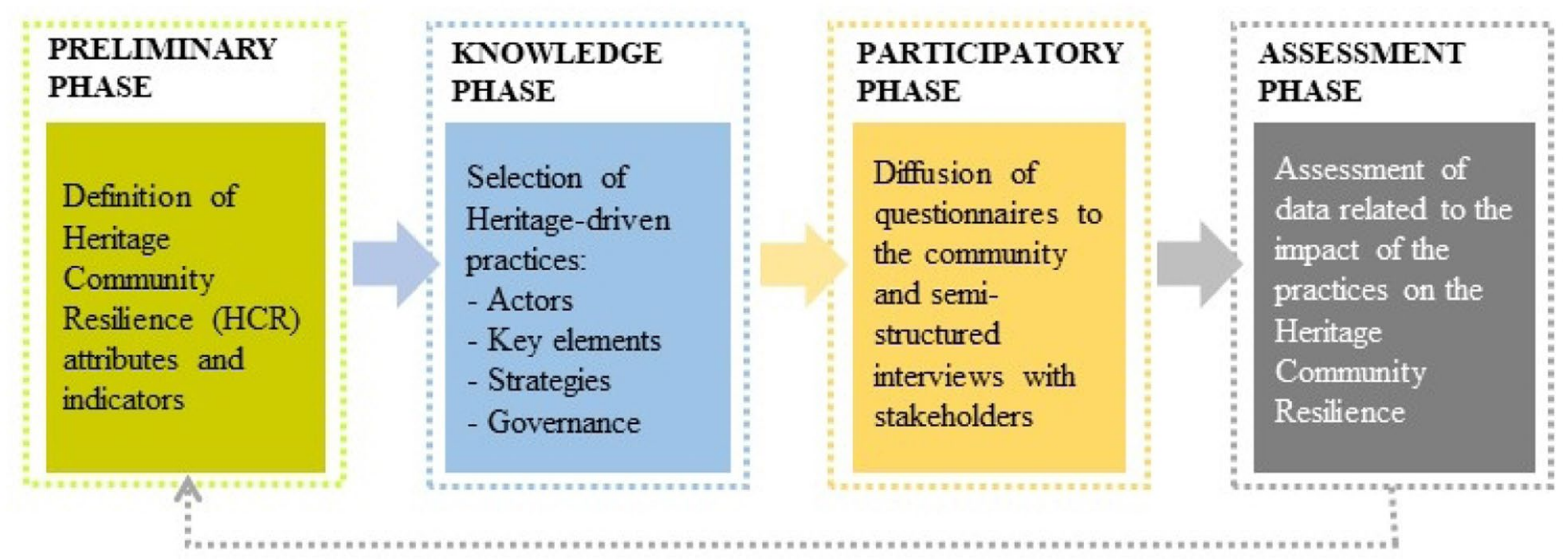
Methodological process

The research methodology was based on the following steps (Fig. 1):

- definition of HCR attributes and indicators, based on sector literature about Heritage Community and Community Resilience, and their explanation in the form of questions to answer with “yes”, “no”, “maybe”;
- selection of heritage-driven practices;
- diffusion of the questionnaire, based on the previous indicators, and its submission to the “community” of the practices through mailing list and paper distribution;
- selection of the stakeholders involved in the practices and their direct interview;
- elaboration of the results, subsequent dissemination and verification.

The first phase defining the Heritage Community Resilience attributes and indicators was carried out analysing, in literature, assessment tools of both the issues Heritage Community and Community Resilience. Regarding the first issue, the Faro Convention Action Plan 2018–2019 (Council of Europe 2018) defines the attributes of what makes a HC. In particular, the Action Plan formulates 12 criteria to self-assess, self-monitor and self-evaluate the activities of the Faro Convention Network good practices. These criteria are inspired by values of social inclusion, human rights and community well-being (Council of Europe 2018).

Unlike the Heritage Community, many scientific studies develop the characteristics of Community Resilience (Maguire and Hagan 2007; Cutter et al. 2008; Berkes and Ross 2013; Chelleri et al. 2016; Rapaport et al. 2018; etc.). Some of them develop real evaluation systems (Magis 2010; Longstaff et al. 2010; Wilding 2011; Maclean et al. 2014; etc.) that vary in relation to the objectives and risks faced by the community.

To provide a framework for the survey, we started from the six attributes of Community Resilience proposed by Maclean et al. (2014), previously tested in Berkes and Ross (2013). These attributes emerge from a collective research in which the authors analysed a series of communities that successfully adapted to rapid and oftentimes crises-driven changes, deducing the key factors for the success.

**Fig. 2:**
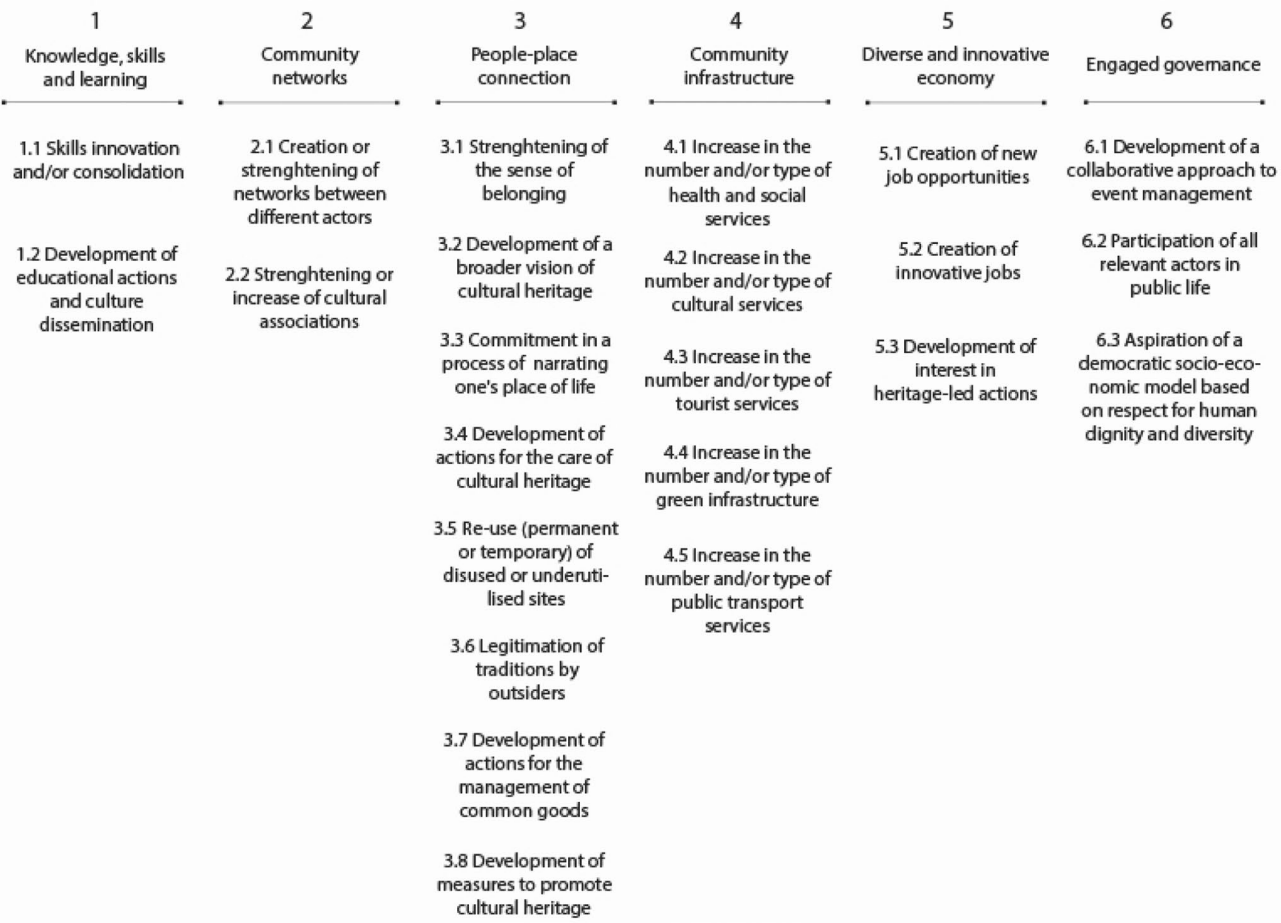
Heritage Community Resilience attributes and indicators

Based on these six attributes, 23 indicators of Heritage Community Resilience were formulated for the construction of a survey questionnaire (Fig. 2). In our research, these indicators are able to assess the resilience of a Heritage Community, so as defined in our conceptual framework, i.e. both an objective and a process in which the community builds, through cultural heritage, its capacity to anticipate and adapt to the challenges and stress factors encountered before, during and after a disaster and/or disruption.

The first attribute concerns “knowledge, skills and learning” defined as “individual and group capacity to respond to local needs and issues” (Maclean et al. 2014, p. 149). These can include practical knowledge but also soft skills like management and communication. In our hypothesis, culture can support and consolidate the process of developing this individual and collective capacity (Fig. 2). The second attribute is about either “community networks” or the existence of collective activities and projects in which community members participate, thus strengthening the links between themselves and creating common interests. In our hypothesis, the diversification of networks and actors is critical in the construction of social capital. At the same time, cultural associations can play a central role inside and outside the Heritage Community. The third attribute is called “people–place connections”. In order to become more resilient, community members must have a “close connection to their biophysical environment” (Ibidem, p. 150), which means they can take advantage of their resources, but they also have to stand for their protection and care. In our hypothesis, Heritage Community Resilience combines adaptive capacity with a strong identity and sense of belonging; cultural heritage can sustain this process in creative and innovative ways. The fourth attribute relates to “community infrastructure”. Inhabitants should have access to a set of services that guarantee their basic needs (water, food, health care, mobility, education, entertainment, etc.). In particular, lifestyles and livelihoods, infrastructure and support services are crucial for the recovery from disturbances and/or disasters (Kulig et al. 2008). In our hypothesis, when measuring this attribute, cultural services as well as tourism services and green infrastructures must be taken into account. The fifth attribute concerns the ability of the community to build a “diversified and innovative economy”. It considers that the survival of the community depends not on a single but rather on multiple resources. At the same time, it recognises the need to keep up with changing market needs, as well as change in general, as an opportunity for new and diverse jobs. Finally, the last attribute concerns the existence of an “engaged governance” with a “genuine participation from relevant private, public and community sector stakeholders” (Maclean et al. 2014, p. 152) in problem solving and in decision-making. In our hypothesis, it implies that the governance systems include both all the actors who wish to be involved as well as those who are directly impacted by the decisions. Moreover, since one of the characteristics of a Heritage Community is the Commitment to principles of human rights in local development processes, it has been included among the indicators (Fig. 2).

After a phase of selection of heritage-driven practices, a questionnaire was elaborated, based on the previous indicators, and disseminated to the “community” of each practice. Each question was formulated as follows: “do you think the practice contributed to…” and completed by each indicator. Three possible answers were offered: “yes”, “no” and “maybe”. The questionnaire was distributed to about 500 people through a general mailing list and paper distribution in places of aggregation.

In addition, during the dissemination of the questionnaire, a series of semi-structured interviews were conducted with the stakeholders. They were representatives of the institutions and the main actors involved in the practices. The 17 interviews lasted between 30 and 90 min, and focused on six main open themes: the origin of the project and the reason of their involvement, the project timeline, the role the interviewee play in the event, the stakeholders with whom the interviewee cooperated, the management of the event, and the results on the territory and on community everyday life. They represented a necessary support for the interpretation of the results obtained through the questionnaire.

For the data analysis concerning the questionnaire, the answers were classified according to the five categories of respondents: institutions (also including administration, trade associations, etc.), actors involved in the event management (creators, organizers, designers, artists, volunteers), partners, such as entrepreneurs and traders, residents in the municipality of the event, residents in the region or occasional tourists. The classification was necessary since not all the actors have equal information about the event. The actors involved in the management of the event have a detailed knowledge of its functioning and experience in decision-making systems, while residents and tourists are better able to express themselves on the sustainable improvements generated by the events on the community.

For data interpretation, graphs were made that show the contribution of each practice to the six attributes, by category of actor (Figs. 7, 8 and 9). A final graph (Fig. 10) summarizes and compares the results of the three previous tables through a prior weighting of the sample, according to the number of responses contained in each category of actors.

## Research context and case studies

**Fig. 3:**
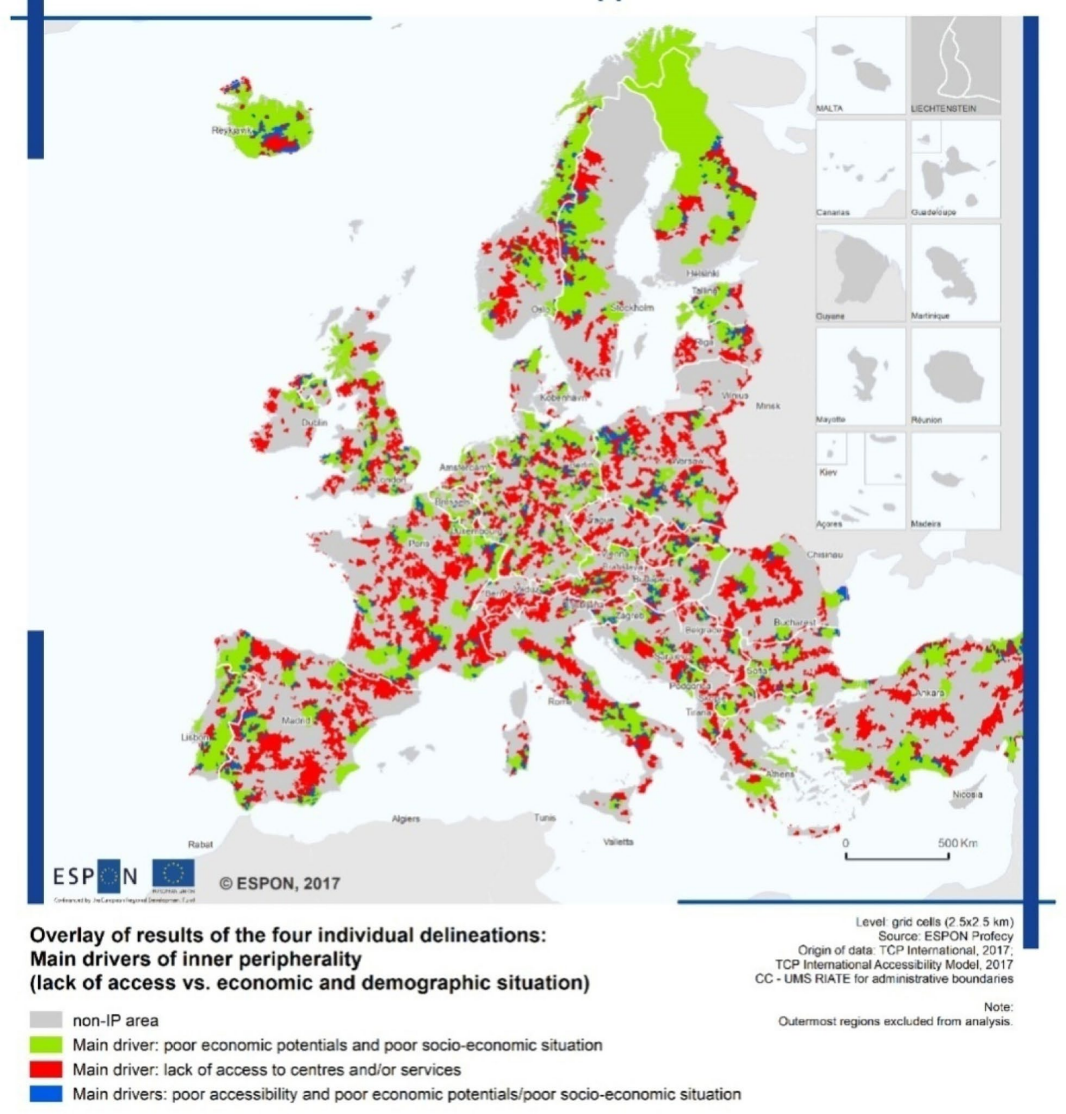
European inner peripheries. Source: ESPON (2017)

The research context is that of the internal European peripheral areas, with particular attention to an area of southern Italy: Alta Irpinia (in the Region of Campania, Italy). This area is composed of 25 municipalities covering 1.118 sq.km., with a population of approximately 65.000 inhabitants. It was selected as one of the 72 pilot areas of the Italian Strategy for Internal Areas (SNAI), launched in 2012 by the Italian Minister for Territorial Cohesion with the main aim of reversing demographic trends. In the classification of the Strategy, this area is composed by peripheries and ultra-peripheries, defined as areas “very diversified within themselves, far from large agglomeration and service centres and with unstable development trajectories but nevertheless with resources that are lacking in central areas, with demographic problems but also highly polycentric and with strong potential for attraction” (Territorial Cohesion Agency 2014). This definition tends to overcome the traditional dichotomy between cities and countryside or between mountain and coastal cities, to underline, in accordance with the European definition of peripheral inner areas, “the degree of disconnection of these areas with neighbouring territories and the network, and not (or not only) their geographical position with respect to the centres” (ESPON 2017) (Fig. 3).

The resilience approach for these areas allows to interpret the dynamics of reaction and adaptation to local and global risks (depopulation, reduction of employment and sustainable land use, landscape degradation—caused, in turn, by hydrogeological, seismic, anthropogenic, environmental risk conditions); at the same time, it allows to define factors, endogenous and exogenous, that can influence these dynamics and can enable and facilitate changes. In addition, this approach makes it possible to define the thresholds of the variables that guide these processes, which in contexts for different aspects very vulnerable – built environment with “simple” qualities, collective memory entrusted to an aged population, natural and agricultural landscape unprofitable, etc.—can lead to trade-offs and cause irreversible changes.

For the purposes of our research, inner peripheral areas represent an interesting laboratory to investigate the role of Heritage Communities for Community Resilience, and more generally to study in greater detail the characteristics of the resilience process, along with the role that the community and institutions can play in it (Pike et al. 2010). European peripheral areas share a set of common characteristics that are both the cause and result of their remote nature (Pezzi and Urso 2016); poor access to services of general interest and to job and education opportunities, limited market access of local actors, emigration of skilled people, ageing population with the need of appropriate infrastructures and services, low accessibility in terms of both transport and communication systems (digital divide), high socio-cultural capital linked to peculiar material and immaterial heritage, high air and water quality. In this context, which has been characterised by high state intervention and exclusion from networks and political power in the decision-making process, the issue of governance is crucial (Herrschel 2012; Pezzi and Urso 2016).

Therefore, remoteness generated both the conditions that have determined the ability of these areas to adapt, which today represent the potential conditions for increasing their resilience. European peripheral areas preserve almost intact their material culture (robustness) and have a “latent territorial capacity”, also linked to their immaterial culture, that offers high potential for innovation (adaptive capacity) (Pinto et al. 2018).

In this cultural context, in recent years, either bottom-up or mixed bottom-up and top-down experiences have been developed and intensified. Many of these, starting from the rediscovery of values of local heritage have experimented the creation of opportunities for work, leisure, networking, research, etc. These experiences are carried out by different actors—residents, returnees, new inhabitants, tourists, volunteers, etc.—who have designed from time to time new community formations; at the same time these are characterized by innovative decision-making and management processes and new interactions with traditional institutions (Magnaghi 2018; Pinto et al. 2018).

This research analyses three cases of cultural and creative practices in the single territorial context of Alta Irpinia (in Campania, Italy). Several issues are weakening this area. First of all, the depopulation process: between 2001 and 2011, the population decreased by 5.8%, exceeding both the regional (1.4%) and the national (2.3%) average for non-core areas (elaboration of ISTAT data, ISTAT 2011). The main cause is the out-migration: people leave the territory to find working opportunities somewhere else. The second weakness, which is connected to the first one, is the ageing of the population. The ‘over 65’ represents about 30% of the population of the area (ISTAT 2011). The third main weakness is the erosion of the cultural capital and the territorial identity, which are mainly challenged by external pressures on local resources: the landscape is frequently threatened by wind turbines, oil drilling and landfill projects. The change in agricultural land use represents for the resilience theory a so-called trade-off, caused precisely by the loss of value of agricultural land.

### Case studies

**Fig. 4:**
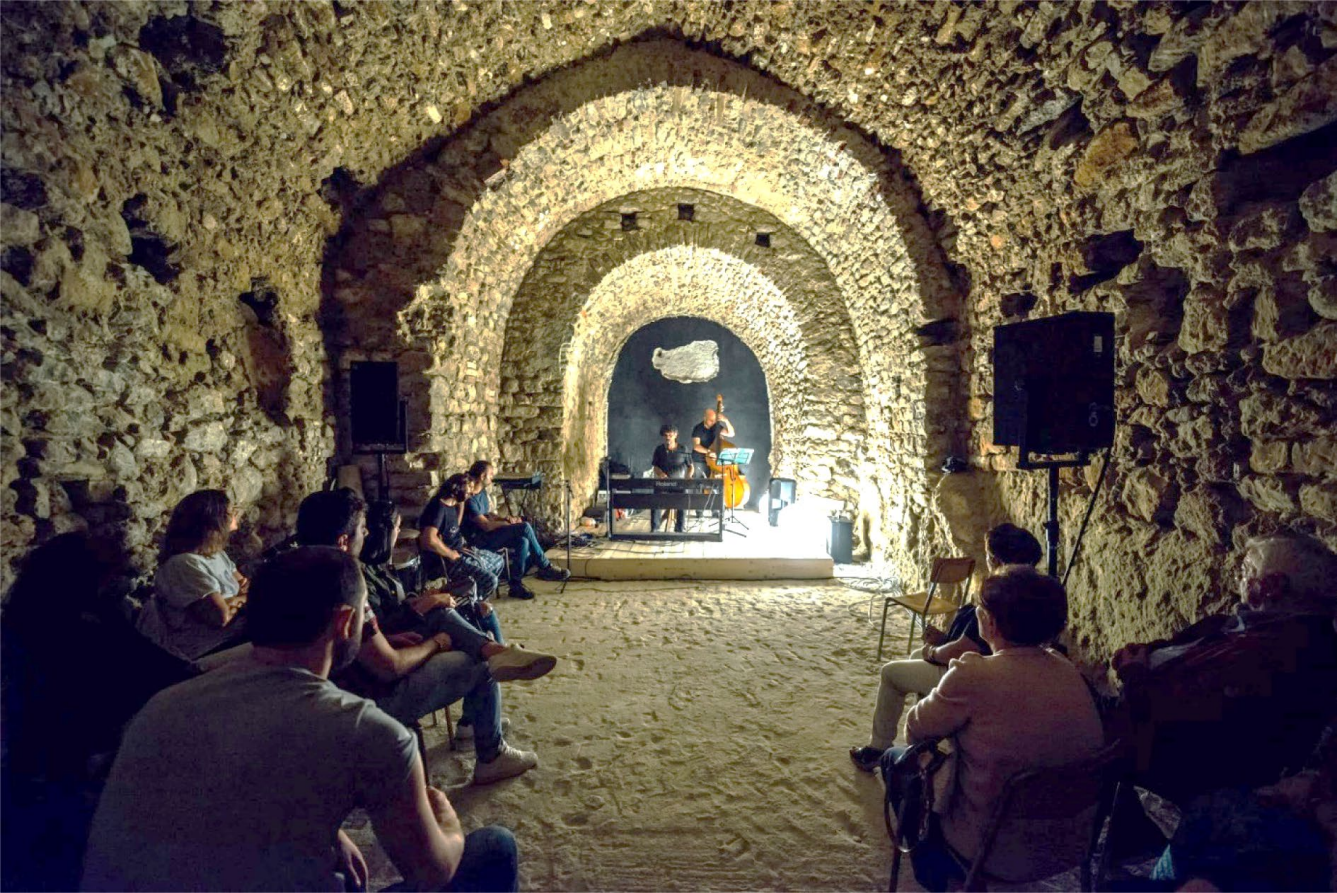
SponzFest: concert in the grotto. ©Giuseppe Di Maio (2017)

The first practice analysed is the Sponz Festival (Fig. 4), which is held every August since 2013, in the Municipality of Calitri. The artistic direction is provided by the singer and composer Vinicio Capossela, who works closely with a local association called “Sponziamoci” to organize the festival. Together with the municipality, they are the main protagonists of the management of the event. Their shared objective is to draw attention to this small village while creating community empowerment. They rely on an original reinterpretation of the local intangible cultural heritage, such as rituals, traditions and narratives. In addition, the program is dense and varied with musical concerts, conferences, film screenings, and artistic performances. It is organized in the oldest part of the village, reusing semi-abandoned public spaces. Part of the programme takes place in five other municipalities in Alta Irpinia. The audience is about 1000 people per day, with peaks that in 2020 reached about 40,000 presences at the final concert (growing from 2013). This is an international audience, even if 70% of the participants come from the Campania Region (data provided by the organizers, who each year elaborate a self-assessment). The economic support is provided by local institutions (municipalities and Local Action Groups), but also in a large part by the Campania Region through European funds.

**Fig. 5:**
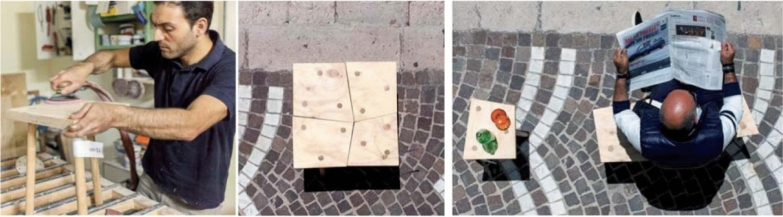
Translations workshop: new design for the ancient “stool” (Andrea Anastasio and his students design). ©e.colonia (2015)

The second case is the Translations workshop (Fig. 5), which takes place in the Municipality of Aquilonia. It is an experimentation of a larger project called “e.colonia”, which aims to create a training and artisanal district, where designers and makers can work together innovating the local artisan knowledge. The organizing group is composed of local architects and academics, who manage the event together with the Local Action Group (LAG), offering financial support. They form groups composed of about 50 people, including local craftsmen, designers, artists and students, who produce prototypes of “rural design” objects in two weeks, inspired by local traditions and skills. The project required the workshop to be annual, so as to create a new artisan network, which could better connect designers to regional traditions, while also opening up new markets for local artisans (Fabbricatti 2017). However, the lack of political support prevented this from happening. The format, which was probably highly ambitious, clashed with the visions of the municipal administration and with a moment of political redesign of the Local Action Groups in Alta Irpinia. As a result, an exhibition of the prototypes and conceptual ideas was organized at the end of the event. In addition, several professionals who had met during the event, held in 2015, have continued to collaborate.

**Fig. 6:**
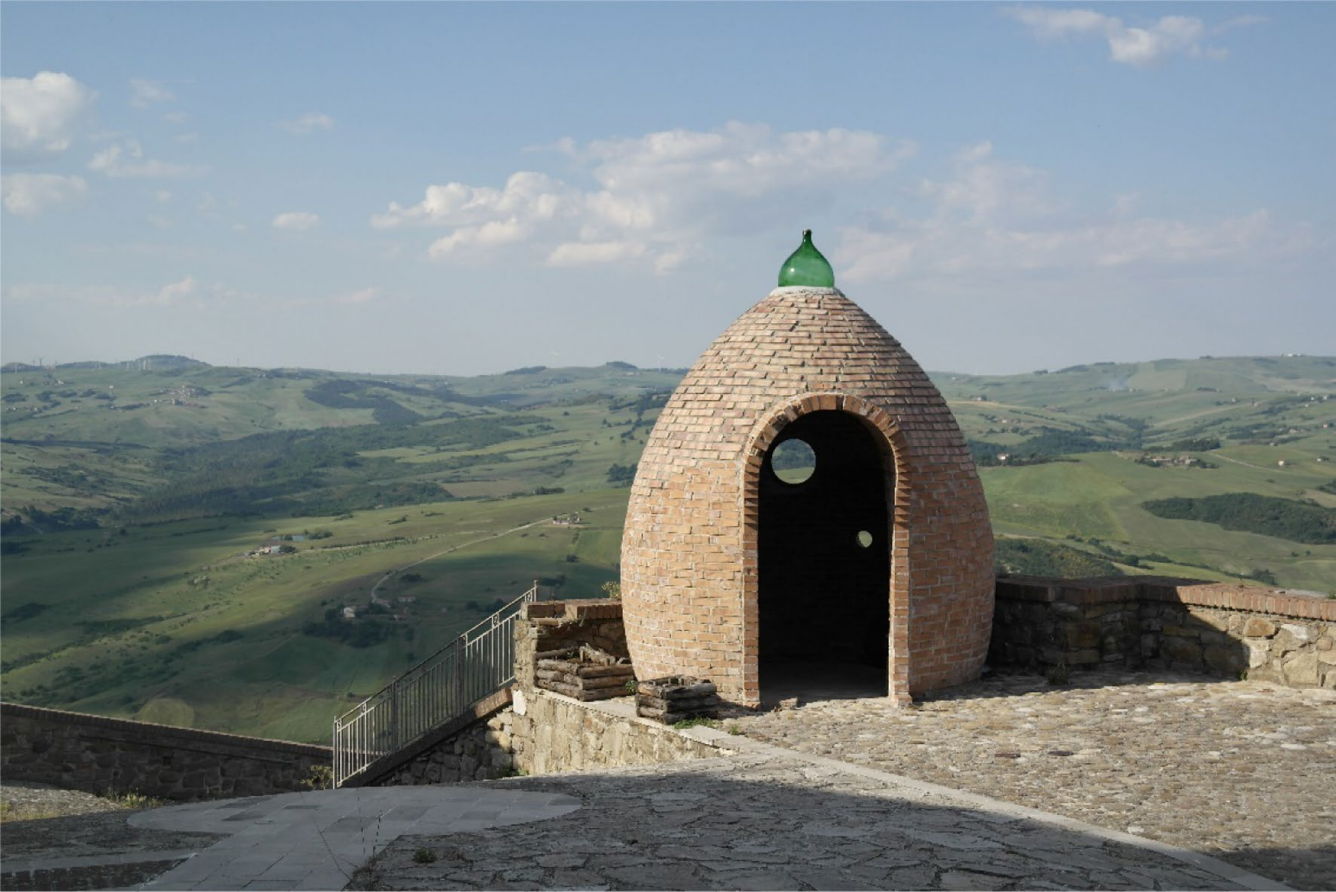
Cairano 7x, 2010. Result of the construction workshop. ©Lucie Boissenin (2017)

The third case is the Cairano 7x festival (Fig. 6). This is also an annual event, organized every summer since 2009. It takes place in the Municipality of Cairano, which is one of the smallest in the territory with only 300 inhabitants. The organizers are both the village association (Pro Loco) and a group of creatives and artists living elsewhere in the province, who create their own association (called “temporary communities” and then “Irpinia 7x”). The objective is to publicize the case of a small village trying to survive by repopulating it for 1 week each year. They also want to reveal the qualities of rural villages, such as air quality, tranquillity and creative inspiration. During this one week, participants can take part in construction workshops, short film and gardening competitions, theatrical performances and activities for children. They can also sleep in the vacant houses of the village. Temporary repopulation brings some years around 500 visitors to the village (Boissenin 2019, p. 116). The series of cultural events fulfil meet the project of the municipality to restore and enhance its built heritage. The economic support was first sourced by local associations and the municipality with the help of a private sponsor, then sustained by European funds. The event management system here is more informal, most of the decisions are taken during the general meetings of the association “Irpinia 7x”, which are moments of conviviality open to both the community and stakeholders.

## Findings

The questionnaire intercepted about 500 people and received 211 responses: 103 for Sponz Fest, which involves a larger audience; 68 for Translations and 40 for Cairano 7x. The “mortality rate” of the questionnaire (about 40%) can be justified primarily by the type of tools that were adopted for its dissemination (email and paper distribution at aggregation points). Moreover, the questionnaire required a commitment in terms of time and concentration, due to the number of questions and their content.

### Sponz Fest

**Fig. 7:**
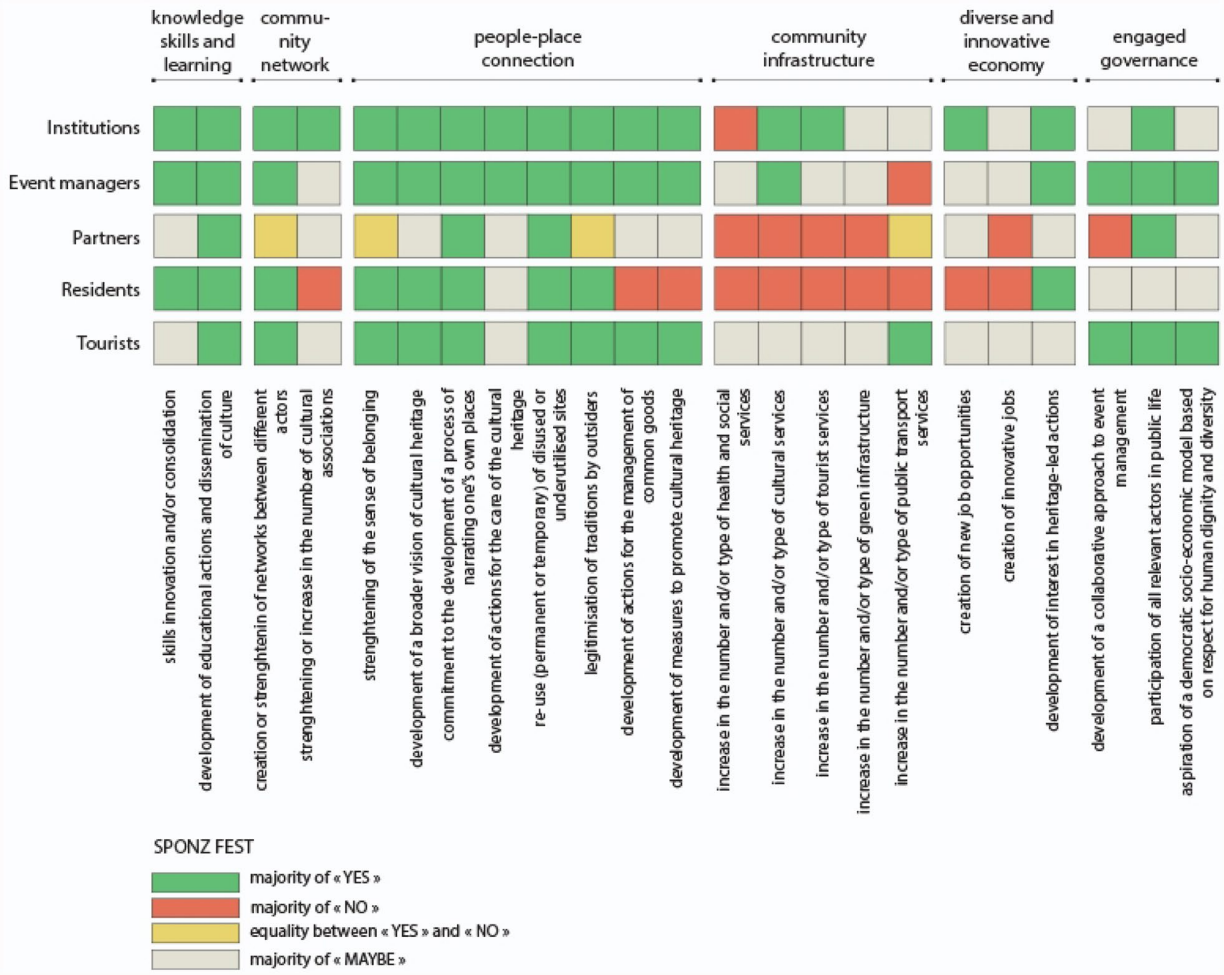
SponzFest: results of the questionnaire

The main positive outcome of the festival regards the “people–place connection” (Fig. 7). The respondents were particularly unanimous about the ability of the event to reuse places and buildings (81% replied yes) and about its contribution to initiate a broader vision of cultural heritage (84% replied yes), including the oral traditions.

The opinions about the “community infrastructure” attribute are more divided. We still obtained 45% of “yes” for the improvement of cultural services and 34% for the touristic ones. The interviews made it possible to understand that the indecision was due to the fact that the positive results linked to the event are temporary and the residents are deprived of these services for the rest of the year. A slight majority of “no” votes prevailed for the indicator about transport services. However, the interviews shed a larger perspective: Sponz Fest was a strong supporter of the local association which has managed to reopen the Avellino—Rocchetta railway line (suspended a few years before due to the lack of users and maintenance). Every year during the festival, a train service was implemented to take participants from other villages to the event, and that contributed to highlighting how the railway line has a touristic potential.

Better results were achieved for the “knowledge, skills and learning” attribute, with a large majority of “yes” (79%) being given by the actors involved in the event organisation. The festival provides significant experience for the volunteers in team management, public reception, communication, both in terms of skills acquired and curricular experience.

The Sponz Fest seems to help build a “diverse and innovative economy” increasing the cultural sector. Half of the respondents believe that the event has reinforced the tendency to develop heritage-led projects, although they question the capacity of these projects to generate new and innovative job opportunities.

Finally, we noticed the strengthening of “community networks” and the building of an “engaged governance”. A diversity of stakeholders were involved, with the participation of six municipalities to the event—which is rare in Alta Irpinia. The organisers, institutions and partners agreed that all the relevant actors were present and played their part in setting up Sponz Fest, with respectively 50, 71 and 67% of the votes.

### Translations workshop

**Fig. 8:**
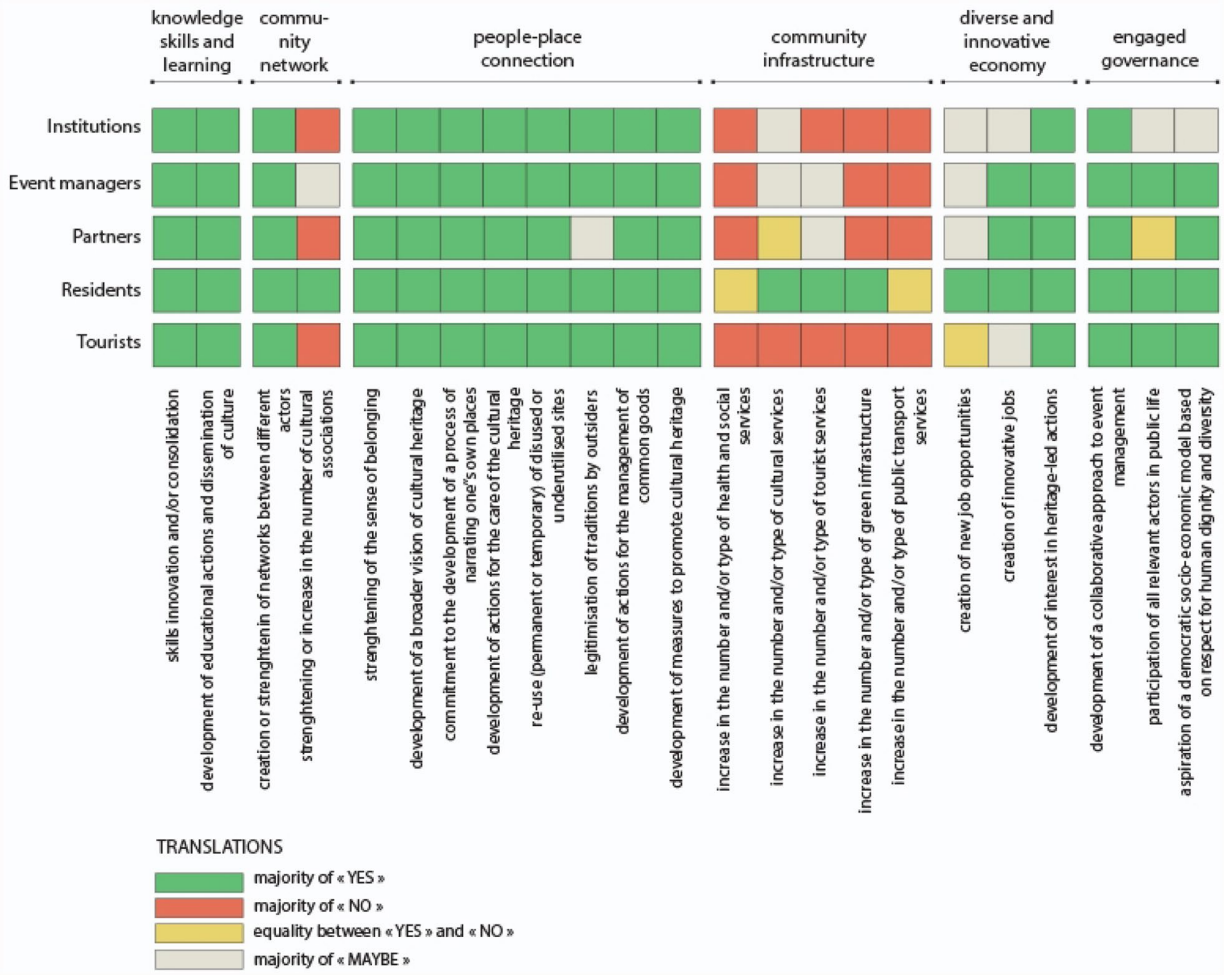
Translations workshop: results of the questionnaire

According to the answers of the questionnaire, the main positive outcome of the Translations workshop was the enhancement of “knowledge, skills and learning” of the involved actors: 100% of the institutions, organisers and participants, and residents said “yes” (Fig. 8). The reinterpretation of artisanal traditions, experienced here, also led to new economic perspectives for the area: a majority of the organisers (53%), partners (80%) and residents (50%) suggested that the workshop could open up new innovative jobs.

On the contrary, the event seemed particularly powerless in relation to the improving of “community infrastructure” with a majority of “no” for 4 of the 5 indicators. The workshop is part of a larger project to reuse the ancient abandoned area of the village of Aquilonia as a design academy. The project for political and financial reasons has yet to be realized. It would have represented an important cultural infrastructure. This result leads to the issue of “engaged governance”. The stakeholders seemed unanimous about the collaborative approach of the event (from 50% for tourists to 100% for the institutions and residents) and generally agreed on its capacity to involve all the relevant actors (from 40% for the partners to 87% for the organizers), but the interviews revealed a more mixed view: the involved actors were mainly from the private sector and we could also notice the reluctance of the municipality of Aquilonia, which led the project promoters to seek the support of other institutions, such as Local Action Groups.

### Cairano 7x

**Fig. 9:**
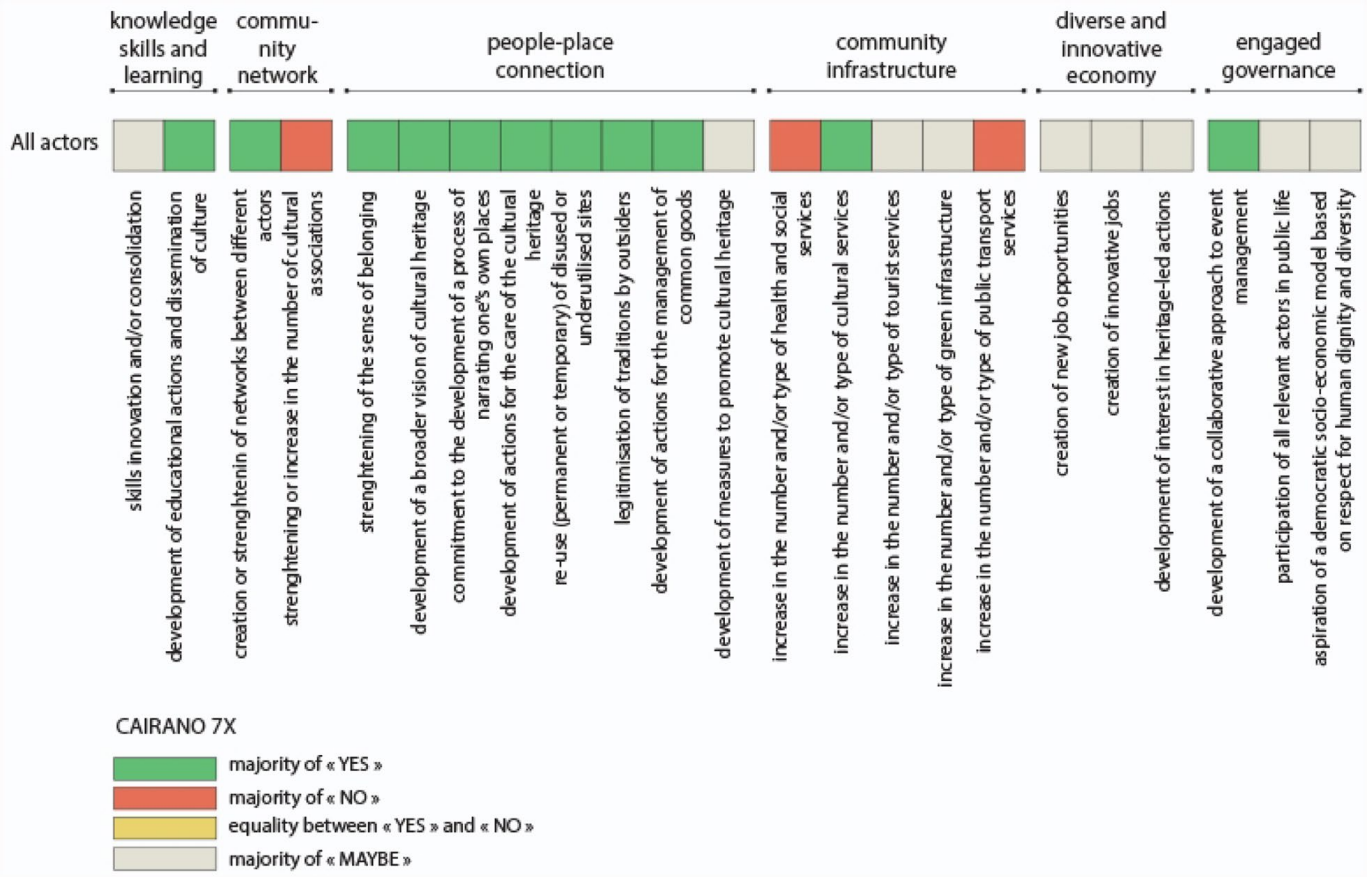
Cairano 7x: results of the questionnaire

The results of the survey about the Cairano 7x festival (Fig. 9) were elaborated considering all the interviewees as a single category of actors, due to the small number of answers obtained from the questionnaire (40).

Like Sponz Festival, the interviewed actors particularly agreed on the ability of the Cairano 7x to reuse the abandoned houses. We have to precise that the cultural practice met with the plan of the municipality to restore its built heritage, a theatre school and new services were created in some abandoned buildings. This can explain the more mixed results about “community infrastructure”, compared to other events, with even 40% of “yes” regarding the green infrastructure, in reference to the creation of an open-air theatre.

Nevertheless, the respondents found it difficult to assess the ability of Cairano 7x to generate a “diverse and innovative economy” (80% replied “maybe”). The interviews helped to note that the opening of the theatre academy brings new career opportunities for the young people from the area; they have access to an education program that did not exist before.

When it comes to the existence of an “engaged governance”, 60% of the respondents agreed on the collaborative approach of the event. However, we need to report some conflicts between the external actors (Irpinia 7x association) and inhabitants, along with their own cultural committee (Pro Loco). Only after a few editions, the inhabitants realized the particular value of their village, but they did not agree with the way it was valued, with the activities responding more to the expectations of tourists than to their own. So, they decided to separate from the first association and create their own events. This conflict allowed the locals to move from a passive behaviour to an active construction of a cultural offer that corresponded to their interests and expectations.

### Cross-reading findings

**Fig. 10:**
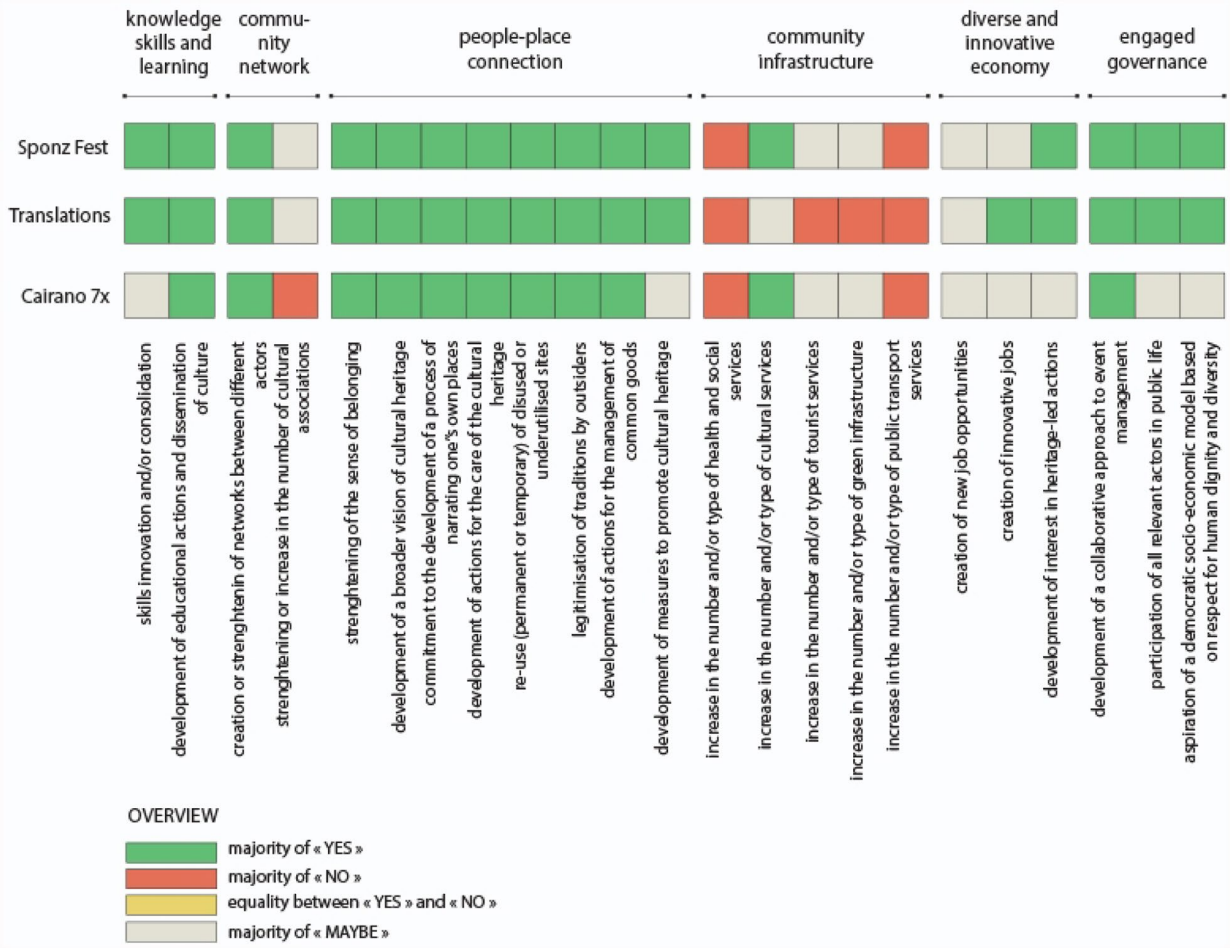
Case studies confrontation: results of the three questionnaire

The cultural and creative practices examined have in common the strengthening of “people–place connection” (Fig. 10). Moreover, the respondents particularly agreed on the attribute of “knowledge, skills and learning” for the ability of the practices to develop educational actions and to enable dissemination of culture (96% on average over the three events). On the contrary, no real impact was noted on “community infrastructures”, with a majority of “no” answers, in particular regarding the mobility infrastructure (68%) and social or health services (54%). Only the cultural infrastructures seemed to be improved in two of the three cases, but most were activated occasionally.

Then, respondents were not able to express themselves on the capacity of the events to generate “diverse and innovative economy”: a majority of “maybe” prevails, caused above all by the seasonality of the events.

A similar situation arises for the indicators related to “community networks”. The responses still converge on the capacity to strengthen existing networks (94%). With the help of the interviews, we know that the three practices did not generate new associations but better articulated the existing ones.

Finally, the apparent consensus on the existence of an “engaged governance” needs to be revisited. For Cairano 7x, a “maybe” majority wins for 2 of the 3 indicators, while all three indicators are green for Sponz Fest and Translations: “development of a collaborative approach to event management” even reaches 72% of “yes” on average over the three practices. The interviews revealed a more mixed picture. While some of the institutions seemed willing to cooperate, others were still very reluctant to engage with the community—a situation shared by the different case studies.

## Discussion

The cross-reference of the results highlights some useful topics for discussion. First, the indicators developed proved effective for the survey and provided a complex view of not only Community Resilience, but also the contribution that the Heritage Community concept can make in its pursuit.

Concerning the effects of the practices examined on building Heritage Community Resilience, the results of the survey demonstrate that a HC is actually emerging in Irpinia, but that the road to building a more resilient community is still long. The practices initiated the community towards a process of acquisition and/or consolidation of knowledge, competence and awareness of its biophysical environment and the care it requires. Moreover, thanks to their involvement in heritage-led practices, the inhabitants discovered that cultural heritage can be a source to build new and creative job opportunities. The examined practices suggested new approaches (innovation of craft knowledge, creation of educational centres, etc.) that diversify the solutions traditionally foreseen for the relaunch of these territories (such as tourism, industrial settlement, etc.), and encourage new economies (SponzFest), start-ups (Translation) and cooperative businesses (Cairano 7x). In addition, through the involvement of different actors and community networks, the practices gave the opportunity to address actions towards building community infrastructures (the reopening of the Avellino-Rocchetta railway line for tourism purposes, the rediscovery of old transhumance routes and green infrastructures, enhancement of cultural and tourist services, of digital technologies, etc.).

With regard to engaged governance, a good cooperation between different types of actors was noted, as well as the participation of a wide and differentiated range of actors. Moreover, some institutions showed a good ability to cooperate in the general interest (Sponz Fest together with six municipalities). However, the poor propensity of some local administrations to engage in a more democratic approach and in vertical collaboration persisted (Translations workshop), as did the existence of conflictual situations between residents and non-residents, due to different interests (Cairano 7x) or to the reluctance of some inhabitants to an innovative approach.

It is also necessary to underline data from the interviews that the indicators did not reveal. The administration of the Municipality of Calitri highlighted that following the SponzFest sales of properties for holiday homes of Italian and foreign visitors were recorded in the historic centre. In addition, to support the survey, quantitative data were collected for some sample indicators. Analyzing the requests for building maintenance and rehabilitation in the municipalities examined, the data show a slight increase since 2015. Although in these municipalities of a few inhabitants, the numbers of requests are low, the data show a constant trend, particularly positive in the municipality of Calitri, which from 2015 shows an almost exponential growth. This data can be referred both to the indicator “development of actions for the care of cultural heritage” of the “people-place connection” attribute, as well as to the “development of interest in heritage-led actions” related to the “diverse and innovative economy” attribute. The latter was also verified by collecting data on the number of the employed population (between 15 and 64 years of age). They are not easy to interpret because of the multiple economic categories they include. Starting from 2011, in fact, Calitri records a negative trend (51.13% in 2011; 49.86% in 2019), while a positive trend is recorded in the municipalities of Aquilonia (50.16% in 2011; 51.20% in 2019) and Cairano (44.54% in 2011; 53.07% in 2019) (ISTAT 2011; Infodata 2019). Direct surveys show, however, for Calitri as well as Aquilonia the creation of some new and innovative jobs, which also reveal synergies between inhabitants of different municipalities. A further survey, through archival data, concerned the number of accommodation facilities, the relative number of stay, and the presence in tourist offices (pro-loco) outside the week or period of the events, which can be referred to the attribute of “community infrastructure”. Also in this case, since 2013 there is a positive trend for Calitri. Aquilonia and Cairano shows a constant trend during the year, that in August sees the population double for the return of natives living abroad, and peak in the days of the festival. Finally, data on the trend of the real estate market since 2013 show an irregular performance with maximum house sales values that remain constant in the municipalities of Calitri and Aquilonia (https://www.agenziaentrate.gov.it). It is useful to underline that this last data excludes, or reduces, the risk of trade-offs, such as the cases of gentrification, that some authors detect by analysing the dynamics of cultural and creative processes (Duxbury and Campbell 2009; Kebir and Crevoisier 2008; Mitchell and de Waal 2009) Other types of trade-offs are pointed out by some authors such as: the risk that the commodification of certain components within the culture could turn a community into ‘a folkloric spectacle’, contributing to the destruction of the very image of rural heritage and to the reproduction of a ‘leisure-scape’ (Mitchell and de Waal 2009); the possibility that certain temporary events can create a gap in the economic and political situation by interrupting the regularity of a virtuous circuit established between the social, economic and political components during the year. To avoid these risks, it is recommended to monitor data over a longer period of time and, as pointed out by Evans (2005), to evaluate the sustainability and distributive equity of cultural and creative practices “seeking better engagement/consultation with local communities to improve ownership of the (cultural) project and (local) benefits” (DCMS 2003, p. 2).

Starting from these findings, it is possible to identify some elements that could positively influence Heritage Community and Community Resilience, in a self-sustaining circuit.

A plurality of actors and competences has proved to be a fertile element for all practices.

The involvement of the local community, internal and external organizers, of external people who had never before visited those places, of both local and external heritage experts was observed in each practice. In this process, the encountering of these different actors helped the unveiling, decoding and enhancing of values to be attributed to cultural heritage, triggering a virtuous circuit of awareness and care.

Building a shared project between the inhabitants and their elected representatives is strategic to achieving CR.

Cultural heritage plays its role as a “federation” in each of the three case studies. Several actors put aside their reticence and personal interests to contribute to a project of collective interest. By learning to work together, many actors begun to rebuild relationships, which are essential for CR. Heritage thus has become a dimension that ‘unites’ and allows for the fertile confrontation among multiple identities. It also allows to highlight the skills and talents of the community, mediating among different points of view and interests, in a perspective of common interest (Fusco Girard et al. 2017).

Building a proactive and responsible role of the inhabitants supports Community Resilience.

From the interviews, we learned that after the event people usually try to continue working on other cultural and creative practices or initiatives. Since the early 2000s and the spread of a cultural movement in Irpinia (Boissenin 2019, p. 113), a succession of cultural practices have led to a growing proportion of local people and new inhabitants becoming active. The community raised its voice, gradually forcing the institutions to take its demands into account. The relationship that exists between the community and place becomes, in turn, a prerequisite for its care and conservation, with direct effects on the reduction of the physical and social vulnerability of the territory.

Recognition of a complementarity between community and institutions is useful for the success of heritage-led practices, and for the objective of Heritage Community Resilience. The positive results achieved in the objectives of Heritage Community and Community Resilience were made possible due to one category of actors taking a step towards the other. The events happened because the creative and management skills of the community met the ability of the institutions to have access to sources of funding and networking. In the case of the Translations workshop, precisely the lack of support from the administration was one of the main reasons for its short duration.

## Conclusions

This paper explored the issue of Community Resilience with the objective of helping to reduce emerging gaps between theory and practice (Stumpp 2013) and at the same time enrich the debate on the contribution of cultural heritage to CR. The paper elaborates a conceptual framework from which to define the perspective of Heritage Community Resilience. This original concept, described in the paper through 23 indicators, can act both as a new target and a process in which cultural heritage supports the building of a community able to prevent, cope with and recover from disturbances. In an evolutionary vision of resilience, culture and cultural heritage can be the key to citizen engagement (idea of common good), as well as to social, environmental, economic and governance innovation.

Through a survey of several heritage-driven case studies, either bottom-up or mixed bottom-up and top-down, based on direct analysis tools, the research tests the Heritage Community Resilience indicators and highlights the characteristics and potential of the concept. The research context is that of Italian inner peripheral areas, proved to be an interesting laboratory for the crucial challenges facing these territories.

The practices analysed demonstrate that although a Heritage Community is actually emerging in Alta Irpinia, and despite the relevance of the issue in these areas, there is a lack of strategies and operational tools aimed at Community Resilience.

From the lessons learned from the case studies, the concept of Heritage Community Resilience imposes shared policies among institutions, residents, sector experts, entrepreneurship, researchers, facilitators, and humanitarian organizations, requiring to equip themselves with participatory management tools and defining a shared framework of governance of cultural policies. In this regard, the further perspectives of this study are to understand the relationship between HCR and Community Based Disaster Risk Management (CBDRM). It is a process in which at-risk communities are actively engaged in the identification, analysis, treatment, monitoring and evaluation of disaster risks, contributing to the reduction of their vulnerabilities and the improvement of their capacities (ADPC 2006; Abarquez and Zubair 2004).

In this direction, a contribution could emerge from the definition of Heritage Community (Council of Europe 2005 art. 2b). It implies collaborative actions of care and maintenance of cultural heritage, affecting both its vulnerability and that of the community. This can trigger a virtuous circuit in which actions on cultural heritage strengthen community cohesion, reduce urban degradation, start urban regeneration actions and employment opportunities. The process for Heritage Community Resilience thus becomes a circular path (Fusco Girard et al. 2017; De Medici et al. 2018) in which actions aimed at Community Resilience contribute to the development and enhancement of the territory in a perspective of a resilient circular city (Fabbricatti and Biancamano 2019).

## Data Availability

The datasets used during the current study are available from the corresponding author on reasonable request.

## Abbreviations

CBDRM: Community Based Disaster Risk Management
CR: Community Resilience
DRR: Disaster Risk Reduction
HC: Heritage Community
HCR: Heritage Community Resilience
